# Human Papillomavirus 16 E2 as an Apoptosis-Inducing Protein for Cancer Treatment: A Systematic Review

**DOI:** 10.3390/ijms232012554

**Published:** 2022-10-19

**Authors:** Dinah Farhanah Jamal, Quratul Ain Rozaimee, Nadila Haryani Osman, Atikah Mohd Sukor, Marjanu Hikmah Elias, Nor Aripin Shamaan, Srijit Das, Nazefah Abdul Hamid

**Affiliations:** 1 Department of Basic Medical Science I, Faculty of Medicine and Health Sciences, Universiti Sains Islam Malaysia, Nilai 71800, Malaysia; 2 Department of Human & Clinical Anatomy, College of Medicine and Health Sciences, Sultan Qaboos University, Muscat 123, Oman

**Keywords:** apoptosis, cell death, E2 protein, human papillomavirus, HPV 16

## Abstract

Human papillomavirus type 16 (HPV-16) is a well-known etiological factor for cervical and oropharyngeal cancers. The E2 protein, the product of an early-transcribed gene in HPV–16, is postulated to cause the death of cancerous cells via p53-dependent and p53-independent pathways. The main aim of the present systematic review was to study the HPV 16-E2 protein as an apoptosis-inducer agent. A thorough search of MEDLINE/PubMed, Science Direct, Scopus, and EBSCOhost databases was conducted for relevant studies on HPV AND apoptosis OR cell death where HPV 16-E2 was involved. The search identified 967 publications. Eleven records dated from 1 January 1997 to 16 February 2022 were found to meet the inclusion criteria and were eligible for data extraction and inclusion. All studies concluded that HPV 16-E2 was able to induce cell death in transfected cells. E2 proteins from the high-risk HPV–16 were able to induce apoptosis through different apoptotic pathways depending on the location of the expressed gene. However, the mechanism was still unclear, and further studies are warranted.

## 1. Introduction

Cervical cancer has emerged as the fourth-most common cancer affecting women with a 6.5% incidence and 7.7% mortality reported worldwide [1]. The major risk of cervical cancer includes human papillomavirus (HPV) infection, smoking, age, and the use of contraceptives [2]. Human papillomavirus (HPV) is classified into low- and high-risk infectious agents depending on their ability to cause malignancies in normal cells. High-risk HPV types 16 (HPV–16) and 18 (HPV-18) are reported to be responsible for HPV-positive tumors with a 70% global prevalence for cervical cancer [3]. It has been reported that in women aged 15 years and older, HPV-16 is the most common genotype, followed by HPV types 52, 31, and 18 [4].

The HPV genome consists of early and late gene regions where gene arrangements influence the sequence of gene expression and controls the infection mechanism of HPV. Each of the early genes designated as E1, E2, E4, E5, E6, and E7 has specific roles in gene expression and infection [5]. HPV can remain in the epithelial basal layer and cause latent infection in the basal cells. Moreover, encapsulated HPV infects the stratified squamous epithelium cells that are exposed through microlesions. Consequently, viral promoters are activated, resulting in expression of E1 and E2 [6]. E1 and E2 genes encode for the protein complex that leads to E1 attachment to the origin of replication, which helps in regulating the transcription of the subsequent viral genome [7,8]. The E4 protein blocks cell apoptosis by binding with cytokeratin and it is believed to play a role in virus release in order to initiate infection of nearby cells. The E5 protein helps promote cell proliferation. The binding of E6 and E7 to p53 and retinoblastoma (pRb), respectively, leading to DNA replication, even in non-dividing cells. The late gene region mainly encodes major and minor structural capsid proteins, such as L1 and L2. capsid proteins enable the complete genome to exit the infected cells as an encapsulated infectious virus [9]. Targeting the major capsid L1 to activate humoral antibody responses has become the major principle in developing the HPV vaccine, which is now commercially available [10]. The bivalent Cervarix^®^ vaccine consists of virus-like particles (VLP) L1 from HPV-16 and HPV-18, while the quadrivalent Gardasil^®^ vaccine consists of VLP L1 from HPV types (6, 11, 16, and 18), thus providing wider protection against HPV [10]. Gardasil 9, a nine-valent vaccine, has been approved by FDA in 2014 and offers protection against HPV-6, 11, 16, 18, 31, 33, 45, 52, and 56 [11]. The 9-valent HPV vaccine provides more protection against HPV compared to the quadrivalent HPV vaccine, is safe, and its cost-effectiveness favours its use in adolescent females [11].

The E2 gene encodes the E2 protein, which is required for other proteins to function, and regulates transcription through interactions with the E1 viral protein in DNA replication initiation [9]. It is involved in cellular gene expression, growth inhibition, and apoptosis [12]. In HPV-transformed malignant cells, the chromosomal integration between HPV and the host cell disrupts the E2 open reading frame, leading to the deduction that the absence of E2 protein expression is a vital step for the deregulation of the cell cycle, and the initiation of carcinoma transformation [13,14,15]. Nevertheless, the HPV-16 E2 protein has the ability to induce apoptosis through more than one pathway, which is through p53-dependent and p53-independent pathways [16,17,18,19]. Both HPV-transformed and non-transformed cells have p53-dependent pathways, whereas HPV-transformed cells only have p53-independent pathways [17]. There are variable opinions among researchers regarding the pathways and proteins involved in E2 apoptotic functions. E2 proteins from high-risk HPV types 16 and 18, which are associated with cancer of cervix, were reported to induce apoptosis [16]. In addition to controlling transcription and viral DNA replication, E2 proteins physically interacted with cellular proteins to have an impact on the biology of the host cell [16]. The involvement of the E2 protein in apoptosis occurred via the p53-dependent and p53-independent pathway, receptor-signaling pathway, and mitochondria-dependent pathway [16,17,18,19,20,21]. Published studies showed that E2 induces apoptosis indirectly, via its effects on the expression of E6 and E7, and directly, via its interaction with p53 [17]. Another mechanism is that there is the binding of E2 to the viral genome, and this can only happen in HPV-transformed cells [17]. E2 possesses multi-functional properties and has a role on the normal viral life cycle of keratinocytes [18]. The viral DNA is amplified and encapsidated by viral structural proteins as the host cell matures until mature virus particles are shed with squames from the epithelial surface [18]. Hence, a systematic review was conducted to evaluate the HPV-16 E2 protein as an apoptosis-inducing agent in both HPV-transformed and non-HPV-transformed cells. This systematic review may pave the way to finding novel methods of using the E2 protein as a potential treatment strategy for HPV infection-related cancer.

## 2. Materials and Methods

A comprehensive search strategy was conducted to assemble the available literature on the apoptosis-inducing function of human papillomavirus type 16 E2 viral proteins. Four well-known research databases were chosen, and search strategies were designed according to Preferred Reporting Items for Systematic Reviews and Meta-analyses (PRISMA) guidelines 2020.

### 2.1. Search Methods for Selection of Studies

#### 2.1.1. Literature Search

A thorough literature search was conducted using four databases: MEDLINE/PubMed, EBSCOhost, ScienceDirect, and Scopus were searched from 1 January 1997 to 16 February 2022. Two investigators (D.F.J and N.A.H) conducted the title and abstract search using keywords chosen for the widest search coverage of any possible studies on the HPV-16 E2 protein but simultaneously retaining the specific objective of the study. The searches were not restricted to any publication year or language.

The following keywords were used for the literature search; “Human papillomavirus 16 E2”, “HPV 16 E2”, 1 or 2 or 3, “Apopto*”, “programmed cell death”, 5 or 6, “cancer”, “malignancy”, “malignant neoplasm”, “neoplasia”, “neoplasm”, 7 or 8 or 9 or 10 or 11, 4 and 7, and 13. The asterisk sign indicates the “wild-card” search technique, where all terms that have the same root word as stated were included in the search process.

Step 1. All results obtained from each search database were imported into a separate individual group in one combined Endnote (X7.0.7; Thomson Reuters) library file, where duplicate results were identified and deleted.

Step 2. Abstract and articles’ keywords were reviewed for eligibility by two of the investigators (D.F.J and N.A.H). Titles and abstracts were thoroughly screened, and irrelevant articles were removed. The following exclusion criteria were applied during screening as a guide to remove some of the results from the study selection: (i) study on types other than human papillomavirus type 16; (ii) study proteins on other than E2 viral protein; (iii) company, clinical trial summary reports, in vivo studies, or in silico studies; and (iv) studies published in languages other than English. Any studies deemed as ambiguous underwent a second screening where full papers were retrieved and carefully re-evaluated based on the inclusion criteria.

Step 3. The following inclusion criteria were applied during screening to finalize the study selection: (i) investigation on high-risk HPV type 16; (ii) involvement of the E2 protein as the subject of interest; (iii) investigation of the viral protein’s function in programmed cell death or apoptosis; (iv) test conducted on cervical cancer cells; (v) original research paper (not reviews or proceedings); and (vi) in vitro studies.

#### 2.1.2. Review of Reference Lists

The references from the finalized articles were screened and examined for any possible related studies. The full-text articles were then retrieved and reviewed to ascertain if they met the inclusion criteria.

### 2.2. Data Extraction

Screened papers that made the cut were reviewed, and information such as first author, year of publication, study outcome, type of cell lines, type of vector or co-factor expressing the E2 protein, and methods to determine apoptosis and levels of observed apoptosis were extracted. No restrictions were made in study design or publication date. A data evaluation form was used as a guide to extract the required information, as shown Table 1.

### 2.3. Risk Assessment

As this review focuses on E2’s apoptotic function, all studies must include an apoptosis assay measurement or cell viability test in the methodology. Once confirmed, further evaluation was conducted on the level of apoptosis and the methods used to measure the level of apoptosis.

### 2.4. Summary Measurement

This review aimed to qualitatively investigate the effect of the HPV-16 E2 protein with respect to apoptosis functions. Thus, meta-analysis could not be attempted due to the study’s exploratory design.

## 3. Results

**Figure 1:**
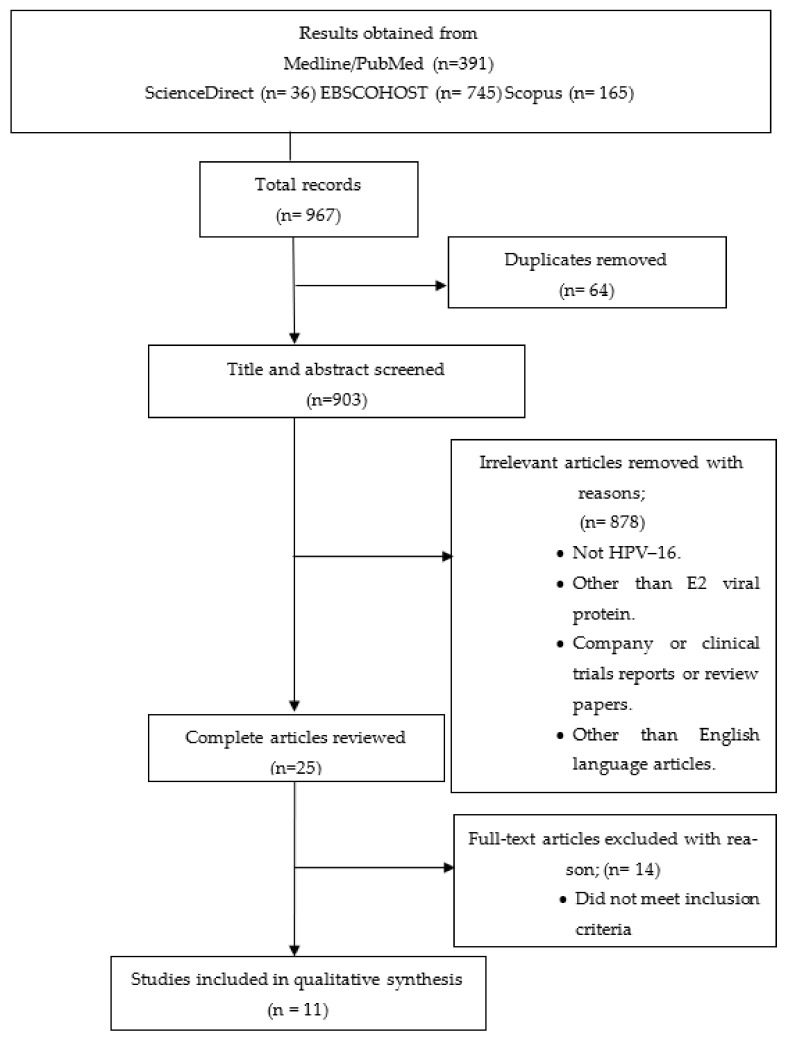
PRISMA used in the methods in the study. Total of records were 967 and a final of 11 studies were included.

A total of 391 records were identified from MEDLINE/PubMed, and 36 from ScienceDirect, 745 from EBSCOHOST, and 165 records from Scopus were selected, resulting in a total of 967 records. Using EndNote X7.0.1 (Thomson Reuters), 64 duplicate results were removed (Figure 1).

Titles and abstracts from the 903 recorded articles were reviewed for their validity. A total of 878 articles were excluded for various reasons, e.g., using proteins other than E2 protein as a cell death causative agent, focusing on different cancerous cells such as head and neck cancers, and different types of high-risk papillomavirus such as HPV type 18 or low-risk papillomavirus such as HPV-11 and HPV-6. Finally, a total of 11 records dating from 1 January 1997 to 16 February 2022 were found to meet the inclusion criteria. All 11 records are experimental study designs involved in testing the HPV–16 E2 protein in vitro. There was only one in vivo study where the combination of radiation treatment and fusion protein E2 was carried out in mice [20]. A total of four studies were conducted in the United Kingdom (UK) [22,23,24,25] and China [19,20,21,26], respectively, two in India [27,28], and another one in Mexico [29]. The two studies in India [27,28] and China [19,20] were carried out by the same team of researchers, presenting the possibility of reporting bias.

The stable or transient expressions of E2 protein in cell lines were the main procedures used to study the protein’s function in vitro. HPV-16 and HPV-18 transformed cervical cancer cell lines, and SiHa and HeLa were the most popular cancer cell lines in these studies. SiHa was used in eleven experiments [19,20,21,22,23,24,25,26,27,28,29], while HeLa was used in five experiments [20,23,24,25,27]. C33A was the most common HPV-negative squamous cell carcinoma used as a comparison; it was used in six of the selected studies [19,20,21,24,26,29]. COS-7, the SV40-transformed monkey fibroblast cell line, has been used in two experiments [23,24] to test for E2 protein expression. MCF-7, an HPV-negative human breast adenocarcinoma, has been used in three experiments [23,25,27]. CasKi and HEK293 have been used in three [23,25,29] and two experiments [25,27] each. Other cell lines such as BMK-16/myc, 778, 877, 915, W12, and B16 were used once in separate experiments [23,24,25,29]. The apoptotic effect was seen in all HPV-transformed cell lines, such as HeLa [20,23,24,25,27], SiHa [19,20,21,22,23,24,25,26,27,28,29], Saos-2 [25], and some of the HPV-negative cell lines such as C33A [19,20,21,24,26,29], MCF-7 [23,25,27], and NIH3T3 [24,25]. Table 2 shows the summary of these cell lines.

### 3.1. Methods to Determine Apoptosis and Cell Viability

The increased or decreased level of cell death was recorded based on the percentage of the cell’s population. We discovered that the most common method for determining apoptosis was by flow cytometry; nine studies used the method [19,20,21,22,23,25,26,27,28]. Six of the studies used either propidium iodide (PI) or Annexin V staining [19,21,22,23,25,27], while another two of the studies used a combination of both [20,26], and one study did not mention the details [28]. Six studies used fluorescence microscopy to inspect cell morphology for apoptosis analysis [19,22,23,24,25,29]. Three of them stated the use of Hoechst 33258 dye to detect apoptotic cells [19,23,24]. Four studies applied the Terminal deoxynucleotidyl transferase dUTP nicked end labelling (TUNEL) assay [19,20,25,29]. Six studies have combined more than one method to determine and measure the level of cell death [19,20,22,23,25,29]. All studies performed an empirical comparison to differentiate cell death caused by apoptosis or necrotic response either based on morphology assessment or by using flow cytometry readings [19,20,21,22,23,24,25,26,27,28,29].

Alongside cell death analysis, three studies used the MTT (3–(4,5–Dimethylthiazol–2–yl)–2,5 Diphenyltetrazolium Bromide) or water-soluble tetrazolium (WST–1) assay (another form of MTT assay) to measure cell viabilities in HPV-16 E2 contained vector transfection [20,22,29]. The cell viability percentage stated was to support the E2 protein apoptotic effect on the cell population. For data collection purposes, the stated value in the records was the average of at least three independent experiments, either for the apoptosis or cell viability test. The *t*-test using mean and standard deviation was the statistical analysis employed in this study.

### 3.2. Vector or Cofactor

Vectors such as recombinant adenoviral plasmid have been used extensively to transfer the E2 gene expression into the cell line of interest. The HPV-16 E2 expression plasmid was produced by cloning the E2 open reading frame gene sequence into EcoR1 restriction sites in pWEB, together with the downstream cytomegalovirus (CMV) promoter to produce pWEB-E2 [23]. This eukaryotic expression vector (pWEB-E2) was used in three separate studies [23,24,25]. Three studies have been identified to use the same pcDNA 3.1 expression system to clone the HPV–16 E2 gene [19,21,26].

The polymerase chain reaction (PCR) was used to amplify the HPV–16 E2 gene before cloning the plasmid of interest. The cytomegalovirus (CMV) promoter and the green fluorescent protein (GFP) were incorporated to improve transcription and assist in protein identification. Two studies co-transfected pCMX-GFP3, which expressed the GFP protein to identify the transfected cells and allow the assessment of cellular morphology [23,24]. Two studies used plasmid pCB6 + p53 for wild-type p53 [22,24], while pCB6 + p53173L represented a mutant p53 plasmid for investigating the p53 interaction [22,24].

In order to control the E2 gene expression in the clone vector, there was a heavy-metal inducible metallothionein promoter used, and the transfection took place upon the induction of cadmium [22]. Other studies used combination treatments by using co-factors such as steroid hormones and radiation to enhance the E2 apoptosis action [20,24].

Among the 11 studies, 6 studies [19,21,26,27,28,29] used Lipofectamine. Only one study [25] used FuGENE 6, Tfx-20, and Tfx-50 as transfection reagents to transfer the expression vector into the cell line of interest. One study [27] applied conventional calcium–phosphate precipitation methods. The summary is shown in Table 3. Information summary of the study’s outcome, type of cell line, plasmid construct, methods to measure apoptosis, and the level of apoptosis is shown in Table 1.

## 4. Discussion

Regarding the present systematic review, we found 11 studies [19,20,21,22,23,24,25,26,27,28,29] on the apoptotic function of HPV-16 E2 that fits our parameters. The re-introduction of HPV-16 E2 protein into the cells was shown to reduce cell growth and promote cell death in serum-starved cells [22]. The E2 protein was shown to increase E6 and E7 mRNA levels. The E7 binds to Rb and releases the free E2F-1 protein, which is believed to induce apoptosis via a p53-dependent pathway.

Admittedly, this systematic review did not include studies on HPV types other than HPV-16. The current systematic review showed that only the E2 from HPV-16 E2 is capable of inducing apoptosis, as reported by Blachon et al. [16] and Parish et al. [17]. It has been suggested that the difference in E2 protein’s ability to induce cell death between low and high–risk HPV types was caused by the intracellular localization of the protein itself rather than its proteomic properties [16,17]. The E2 protein from low-risk HPV type 6 and HPV type 11 remained in the nucleus, while the high-risk E2 protein was present in both the nucleus and cytoplasm. The accumulation of E2 in the cytoplasm activates the caspase 8 cascade, which then leads to apoptosis [16].

There was a report that highlighted the difference between low and high-risk E2 in terms of p53 interaction [17]. The inability of the low-risk E2 protein to bind with p53 fails to induce apoptosis. As for the high-risk E2, E2-p53 binding is required in both HPV-transformed and non-transformed cells in order to induce apoptosis. However, it was suggested that there is a second pathway involved specifically in HPV-transformed cells, where the E2 protein is capable of inducing apoptosis through its interaction with the viral genome E6 or E7 [16].

In another study, the HPV-16 E2 was expressed in a variety of cell lines and showed that the protein was able to induce apoptosis in both HPV-transformed cells and in at least two non-HPV transformed cell lines, C33A and COS–7 cells [24]. The author concluded that apoptosis was p53-dependent and did not require DNA–E2 protein binding. Additionally, the same author in the following year reported that steroid hormones, estrogen, and progesterone could be used to enhance the apoptotic effect induced by the HPV-16 E2 protein [30]. The interaction between E2 protein and p53 was also supported by experiments carried out by Brown et al. [31]. The apoptosis event was measured in both HeLa cells and Saos-2 cells; it was observed that the E2–p53 interaction caused the downregulation of HPV DNA.

There was also a study that succeeded in inducing cell death in non-transfected bystander cells using the modified herpes simplex virus VP22 fusion protein with E2; pVP22-E2 [23]. The expressed VP22-E2 protein was shown to cause apoptosis in infected tumors in a concentration-dependent manner. This finding suggests the possibility of using VP22–E2 as a post-surgical treatment to eliminate remaining tumor cells. However, the further elucidation of this possibility needs to be validated.

It has been reported on the apoptotic role of the E2 protein in radiation treatment in vivo and in vitro [20]. The HPV-16 E2 gene, which was cloned into the novel oncolytic adenoviral M5, was demonstrated to increase the cytotoxic effect on transfected cells in vitro. In vivo, the M5 appeared to enhance the efficacy of radiation treatment in the mice tumor model. Although the exact mechanism between M5-E2 and radiation was not explained, the author suggested a connection to the death-receptor-signalling pathway as caspase 8 activity increased [20].

Despite prior evidence of HPV-16 E2 inducing a high apoptosis rate, a study indicated that there was prolonged stable expression of E2 proteins in human keratinocyte cell lines (HaCaT) that survived the apoptotic effect, providing first insight into E2 protein’s role in malignant transformation after infection [18]. Researchers continued to investigate other possible protein interactions in the apoptosis pathway, and it has been reported that the hyperactivation of caspase-8 and caspase-3 caused by the overexpression of high–risk HPV was mediated by cellular–FLICE (FADD-like IL–1β-converting enzyme)–inhibitory protein (c-FLIP) [19].

Additionally, one study showed that the globular heads of the C1q receptor (gC1qR) play a role in inducing apoptosis in C33A and SiHa cervical cancer cells [21]. gC1qR is a protein embedded in the outer mitochondrial membrane, which is known to mediate many biological responses, including the initiation of apoptosis [32]. The study found that in human cervical squamous carcinoma samples showed significantly lower expression of the HPV-16 E2 and gC1qR genes than non-cancerous cervix samples. In addition, when gC1qR small-interfering RNA (siRNA) was added, the gC1qR gene expression, mitochondrial malfunction, and cellular death were greatly elevated in C33a and SiHa cells that had been transfected with a vector encoding HPV-16 E2. These findings support a mechanism whereby gC1qR exerts a significant influence in HPV-16 E2-induced human cervical squamous carcinoma cell apoptosis via a mitochondria-dependent pathway [21].

C33A cells are HPV-negative cell lines that express mutated p53 [33]. The p53-dependent apoptosis action was deduced when no cell death was observed in HPV-16 E2 protein-infected cells unless it was co–expressed with p53 [24]. COS-7 did not undergo E2-induced apoptosis, thus making it an ideal choice to compare the tagged protein expression in vitro [23]. Saos-2 is a p53-null cell line and it is more likely to be used when the study investigates the effect of p53 in the presence of the E2 protein [25].

In terms of techniques used to measure apoptosis, PI/Annexin V staining via flow cytometry was able to provide a quantitative analysis of the proliferation and condition of the transfected cells where the exact population of cells’ condition could be quantified [34]. Apoptosis analysis via the TUNEL assay generally produces lower readings as it cannot detect late apoptotic cells that have detached. On the other hand, internucleosomal DNA fragmentation detected by the TUNEL assay can be caused by non-apoptotic cells, including necrotic cell death, cells undergoing DNA repair, and cells damaged by other forces, which could lead to the overestimation of reading [35]. Therefore, PI/Annexin V flow cytometry is more accurate compared to the other apoptosis assays employed in the studies. PI/Annexin V staining is capable of differentiating the cell lysates into early and late apoptosis phases, allowing the investigator to obtain data that are more accurate. In comparison of five studies that used both SiHa (HPV type 16) and HeLa (HPV type 18), no significant difference was found between the levels of apoptosis caused by different HPV strains [20,23,24,25,27].

## 5. Conclusions

In addition to controlling viral gene expression, the HPV E2 protein is necessary for viral replication. The current systematic review showed that HPV-16 E2 induced apoptosis in both HPV-transformed and non-HPV-transformed cells. The tumor-suppressor gene p53 is responsible for the human genome stability and has the potential to repair DNA. The E2 gene interaction with p53 is vital in non-HPV-transformed cells but not in HPV-transformed cells. The involvement of E2 gene in the p53-dependent and p53-independent apoptotic pathways makes the E2 gene a suitable candidate for the therapeutic targeted gene not only for cervical cancer but also potentially for other cancer types as well. Nevertheless, further studies on larger sample size are warranted to elucidate the apoptosis mechanism and the apoptotic effect of E2 protein.

## Figures and Tables

**Table 1:** Information summary of study outcome, type of cell line, plasmid construct, methods to measure apoptosis, and level of apoptosis.

First Author (Year) (Ref)	Study Outcome	Vector or Cofactor Used	Method to Determine the Apoptosis			Cell Viability Test	Level of Apoptosis Achieved	Cell Line Used
			Flow Cytometry	Fluorescence Microscopy	TUNEL Assay			
Wang et al., (2011) [19]	Overexpression of E2 induces apoptosis in both negative and positive cervical cancer cells leads to hyperactivation of caspase-8 and caspase-3. There is an interaction between HPV16 E2 and c_FLIP from immunoprecipitation experiments. Moreover, c-FLIP and a caspase-8 inhibitor protect cells from HPV16 E2-mediated apoptosis. Overexpression of c-FLIP rescues cervical cancer cells from apoptosis induced by HPV16 E2 protein expression.	pEGFP-C1 pcDNA3.1-V5-His pcDNA-cFLIPS and pcDNA-cFLIPL	Yes	Yes	Yes	Yes	Decrease cell viability 48 h post-transfection (*p* < 0.01). When compared with the control plasmid-transfected cell, E2-transfected SiHa cells exhibit a massive induction of apoptosis detected. The increase in early apoptosis (annexin V positive cells) depends on the anti-Fas antibody concentration in the E2-GFP-transfected cells. The increase in apoptosis induced by E2-GFP over the GFP control was 1.5–3.5-fold, proportional to the anti-Fas concentration.	SiHa C33A
Wang et al., (2011)[20]	Adenovirus with E2 gene dampens the expression of E6 and E7 in cervical cancer cells and has reduced toxicity compared with treatment using wild-type Adv5. Combined treatment with radiation increases potency significantly.	Adv5-E2 (M5)	Yes	No	Yes	Yes	Increased potency (*p* < 0.01)	SiHa HeLa C33A
Chen et al., (2014) [21]	HPV 16 E2-induced apoptosis in cervical cancer cells via a mitochondria-dependent pathway involving gC1qR gene expression.	pcDNA 3.1 pcDNA-HPV-16 E2 pcDNA-HPV-16 E2 mutant (mut)	Yes	No	No	No	(Early and late apoptotic region)	SiHa C33A
Sanchez-Perez et al., (1997) [22]	E2 production led to increase in free E2F-1; induce the apoptotic cell death via the p53-dependent pathway.	pMEP-4 pGL2NA(mdm2) pCB6+p53 pCB6+p53173L	Yes	Yes	No	Yes	Cell viability decreases to 40% with E2 plasmid and cadmium.	SiHa
Roeder et al., (2004) [23]	E2 can be transported into target cells as VP22 fusion protein and induce apoptosis when produced in mammalian or bacterial cells.	pWEB-E2 pHisVP22-E2 pCMX-GFP3	Yes	Yes	No	No	40% of cells in G_0_ state.	HeLa SiHa MCF-7 B16 COS-7 CaSki 873F W12
Webster et al., (2000)[24]	HPV 16 E2 is able to induce cell death even in the absence of other viral proteins and is p53-dependent. Apoptosis occurred in both HPV-transformed and non-transformed cells.	pCB6+p53 pCB6+p53173L pWEB pWEB-E2 pWEB-E7 pWEB-E2 + pWEB-E6 pWEB-E7 + pWEB-E6 pWEB-E2_DBDm_, pWEB-E2Ct pCMX-GFP3	No	Yes	No	No	~35% apoptotic cells happened maximally after 30 h and with 300 ng of expression plasmid.	HeLa SiHa 866 873 877 915 C33a COS-7 808F NIH 3T3
Green et al., (2007) [25]	A recombinant VP22-E2p52m induces apoptosis in HPV-transformed cells with high specificity.	pWEB-E2 pWEB-E2p53m	Yes	Yes	Yes	No	E2 and E2p52m induced significant levels of apoptosis in HPV-positive cell lines. In the HPV-negative cell lines Saos-2, MCF-7, and NIH3T3, the wild-type E2 protein induced apoptosis whereas the E2p53m protein failed to induce apoptosis. Wild-type E2 induced apoptosis in 808F and 873F cells, two examples of normal non-transformed cervical fibroblast cells, whereas E2p53m failed to induce apoptosis in these cells.	866 879 CaSki W12 SiHa HeLa 808 778 873F 808F Saos-2 MCF-7 NIH3T3 Cos-7 HEK293 873
Gong et al., (2021) [26]	HAX-1 plays a crucial role in HPV 16 E2-induced human cervical squamous carcinoma cell apoptosis in a mitochondrial-dependent manner as the expression of the HAX-1 gene significantly increased in cervical carcinoma tissues compared to noncancerous cervix tissues. HAX-1 reduces ROS production, restores the mitochondrial membrane potential, and maintains the integrity of the mitochondrial structure and morphology in C33A and SiHa cells.	pcDNA 3.1	Yes	No	No	No	HPV 16 E2-protein-induced apoptosis in SiHa (HPV 16-positive) and in C33A cell (HPV 16-negative). However, cells that are treated with HAX-1 vector alleviate the HPV 16 E2-induced apoptotic morphology change. Cells transfected with the HPV 16 E2 vector and HPV 16 E2 vector + HAX-1 mut vector shown an increase in the number of cells in the Q2_LR and Q2_UR regions (the region where the early and late apoptotic cells were distributed) when compared with the plain medium.	SiHa C33-A CRL2614
Prabhavathy et al., (2014) [27]	E2 enhances NF-ĸB activation induced by TNF-α in both non-tumor- and tumor-derived epithelial cell besides potentiating STAT3 transcriptional activity induced by TNF-α in HEK293 cells. E2 increases the expression of RelA and its transcriptional activation and retention of E2 was observed in the nucleus with significant interaction with RelA upon TNF-α treatment. Transfection with shRNA-RelA or pretreatment with STAT3 inhibitor had a negative effect on the ability of E2 to enhance TNF-α-induced NF-ĸB activation. Inhibition of STAT3 activation enhanced E2-induced apoptosis, whereas parallel activation of NF-ĸB and STAT3 by the combined action of E2 and TNF-α increased the expression of their common targets, leading to a decrease in E2-induced apoptosis.	pCMV/pCMV-E2	Yes	No	No	Yes	When endogenous RelA was intact, E2 (scr + E2) or TNF-α + scr + pCMV alone induce apoptosis indicated by the increased sub-G_1_ population (47 and 29% respectively), in comparison with the basal level of sub-G_1_ population (17%) while the extent of apoptosis induction was less in their combination (26%) than with their individual treatment. Cells transfected with sh-RelA showed an increase in apoptosis compared to those with intact RelA (24 vs. 17%). E2-transfected cells when co-transfected with sh-RelA had less apoptotic population than those having intact RelA (39 vs. 47%). RelA inhibition in E2-expressing cells treated with TNF-α showed an increase in apoptosis compared to those with intact RelA (32 vs. 26%). Expression of E2 or treatment with TNF-α induced apoptosis, as evidenced by the presence of 37 and 31% of sub-G_1_ population of cells, respectively, whereas it was only 18% when E2-transfected cells were treated with TNF-α. Pre-treatment with STATTIC-induced apoptosis (35% sub-G_1_). E2 or TNF-α induces apoptosis but if the two are together, the cells are rescued from apoptosis and this effect is likely due to the increased expression of the target genes of NF-ĸB and STAT3-like cyclin D1, c-Myc, surviving and Bcl-2 that provide survival advantage to the cells.	HEK293 HaCaT SiHa HeLa MCF7
Prabhavathy et al., (2015) [28]	Re-expression of E2 expression with TNF-α treatment resulted in an increase in the expression of anti-apoptotic Bcl2 (B-cell lymphoma 2) protein and other pro-survival genes such as cyclin D1 (cyc D1), survivin, and hTERT (human telomerase reverse transcriptase).	pCMV/pCMC–E2	Yes	No	No	Yes	TNF-α treatment alone showed an early apoptotic population of 30% and E2-transfected cells with the highest of about 38% whereas their combination reduced the E2-induced effect to 29% with no significant difference seen in both live and late apoptotic populations among them in cells without TNF-α treatment. In SiHa cells, rather the E2 + TNF-α combination significantly down the apoptosis induced by E2 (28%) or TNF-α (22%) to 18% but with a noticeable increase in G0/G1 population (53%) compared with either E2 (45%) or TNF-α (48%) alone. E2-induced apoptotic tendency shifted towards senescence in the presence of TNF-α by arresting cells at both G0/G1 and G2/M phases, thus enhancing cell survival.	SiHa
Bermudez et al., (2009) [29]	HPV 16 E2 protein is able to repress the expression of E6 and E7 oncogenes therefore inhibiting cell growth and to promote cell death by apoptosis both in human and murine HPV 16- transformed epithelial cells. Furthermore, it also has its anti-tumor effects in vivo in immunocompetent mice. Therefore, the use of HPV E2 protein in the prevention and treatment of HPV-associated cancers is proven clinically relevant.	pCMVp16-E2	No	Yes	Yes	Yes	Several changes were occurred in the plasma membrane of the HPV 16-transformed epithelial cells after the cells were transfected with the HPV 16 E2 gene including loss of cell-to-cell contact, cytoplasm shrinkage, as well as DNA ladder and small plasma membrane blebs within the cells. The cell growth rate and viability also decreased.	SiHa BMK-16/myc CaSki C33-A

**Table 2:** Summary of cell lines that have been used in 11 studies. Stated together is the cell line apoptosis response after HPV-16 E2 expression.

Cell Line	Description	Study Involved	Apoptotic Effect
SiHa	HPV-16 transformed cervical carcinoma	[19,20,21,22,23,24,25,26,27,28,29]	Yes
HeLa	HPV-18 transformed cervical carcinoma	[20,23,24,25,27]	Yes
C33A	HPV negative from cervical carcinoma	[19,20,21], [24] *, [26], [29] *	Yes
HEK293	Human embryonic kidney	[25,27]	Yes
COS-7	Monkey kidney fibroblast	[23,24]	No
MCF-7	Human breast adenocarcinoma	[23,25,27]	Yes
NIH3T3	Mouse embryo fibroblast	[24,25]	Yes
BMK-16/myc	Murine c-*myc-1* gene	[29]	Yes
866	HPV-16-transformed cells	[24,25]	Yes
B16	Murine melanoma	[23]	Yes
CasKi	Cervical epidermoid carcinoma	[23,25,29]	Yes
778	HPV-18	[25]	Yes
808F	HPV negative cells, normal non-transformed cervical fibroblast cells	[24,25]	Yes
873	HPV-18	[24,25]	Yes
873F	HPV negative cells, normal non-transformed cervical fibroblast cells	[23,25]	Yes
877	HPV-18, 45	[24]	Yes
915	HPV-16	[24]	Yes
W12	HPV-16 human cervical keratinocyte	[23]	Yes
Saos-2	HPV negative	[25]	Yes

* Failed to induce apoptosis.

**Table 3:** Summary of the transfection reagent used throughout the studies.

Transfection Reagent	Company	Author
Tfx-20	Promega, Madison, WI	[25]
Tfx-50	Promega	[25]
FuGENE 6	Roche	[25]
Lipofectamine	Invitrogen	[19,26,27,28,29]
Lipofectamine	Life Technologies	[21]
Calcium phosphate	NV	[27]
Electroporation	NV	[22]

NV: Not verified.

## Data Availability

Not applicable.
